# Prognostic value of B7-H3 expression in patients with solid tumors: a meta-analysis

**DOI:** 10.18632/oncotarget.21114

**Published:** 2017-09-21

**Authors:** Xianyun Zhang, Chuntao Fang, Guangbo Zhang, Fujin Jiang, Lei Wang, Jianquan Hou

**Affiliations:** ^1^ Department of Urology, First Affiliated Hospital of Soochow University, Suzhou, Jiangsu, China; ^2^ Department of Urology, The Affiliated Huai'an Hospital of Xuzhou Medical University, The Second People's Hospital of Huai'an, Huai’an, Jiangsu, China; ^3^ Department of Gynaecology, The Affiliated Huai'an Hospital of Xuzhou Medical University, The Second People's Hospital of Huai'an, Huai’an, Jiangsu, China; ^4^ Clinical Immunology Laboratory, First Affiliated Hospital of Soochow University, Suzhou, Jiangsu, China

**Keywords:** B7-H3, solid tumor, survival, prognostic biomarker, meta-analysis

## Abstract

Increasing evidence suggests B7-H3 is aberrantly expressed in various cancers, though its prognostic significance in solid tumors remains controversial. We therefore performed a meta-analysis to clarify the prognostic value of B7-H3 expression in human solid tumors. The PubMed and Embase databases were searched, and 28 studies involving 4623 patients were ultimately included in the analysis. Hazard ratios (HRs) with 95% confidence intervals (CIs) were utilized as effect estimates to evaluate the association between B7-H3 expression and overall survival (OS), progression-free survival (PFS) and recurrence-free survival (RFS). The pooled results showed B7-H3 was associated with poor OS (HR = 1.58; 95% CI: 1.32–1.90; *P* < 0.00001) and PFS (HR = 1.67; 95% CI: 1.05–2.65; *P* = 0.031), but not RFS (HR = 1.17; 95% CI: 0.89–1.53; *P* = 0.267). These results suggest B7-H3 is a negative predictor of OS and PFS in patients with solid tumors. B7-H3 may thus be a useful prognostic biomarker and therapeutic target for human solid tumors. However, further studies will be needed to more precisely determine the prognostic value of B7 H3 expression.

## INTRODUCTION

Cancer has been the leading cause of worldwide mortality for many years and is a global health problem [1]. Despite tremendous advances in recent decades with regard to diagnostic techniques and therapeutic options, the prognosis of many cancers remains poor, especially in late stages. Clinicopathologic characteristics such as pathological grade and clinical stage are the main prognostic factors currently being used, but these parameters are often not reliably predictive of individual clinical outcomes. Therefore, identification of novel tumor biomarkers that can better predict cancer progression and prognosis is of vital importance.

B7-H3 (B7 homolog 3 protein) is a transmembrane glycoprotein and a member of the B7 family of proteins that was first described during analysis of a human dendritic cell (DC)-derived cDNA library [2]. B7-H3 has a critical function in antitumor immune responses [3], and evidence shows that B7-H3 possesses both co-stimulatory and co-inhibitory capacities in different tumor contexts. In addition, B7-H3 plays a non-immunological role in promoting cancer invasion and progression.

Overexpression of B7-H3 has been described in several cancers, and it was shown that B7-H3 expression is associated with clinicopathological factors and with prognosis in these cancers [4–13]. In some tumor types, high expression of B7-H3 has been linked to a poor prognosis, while in others the opposite relationship has been observed. Wu et al. [12] reported that B7-H3 expression correlates with a favorable prognosis in gastric cancer patients and suggested B7-H3 may promote an immune response. By contrast, Sun et al. [14] found that B7-H3 expression in non-small-cell lung cancer (NSCLC) correlates inversely with the number of tumor-infiltrating lymphocytes, which has the effect of promoting lymph node metastasis. Similarly, B7-H3 expression was identified as a poor prognostic factor in prostate cancer [15]. Thus, reports on the ability of B7-H3 to predict outcomes in various cancers are inconsistent. We therefore conducted a meta-analysis to assess the prognostic value of B7-H3 expression in cancer patients.

## RESULTS

### Characteristics of identified studies

**Figure 1:**
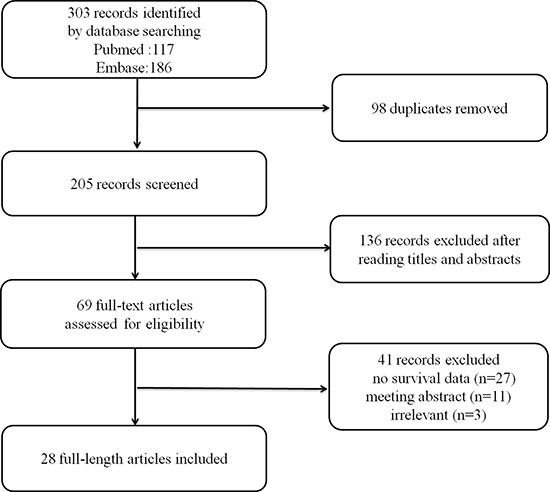
Flow diagram for the selection of studies in the meta-analysis

Twenty-eight studies published between 2007 and 2017 were included in our meta-analysis (Figure 1). A total of 4623 participants were analyzed to evaluate the relationship between B7-H3 expression and the prognoses of various tumors. Of this group, 3170 patients were positive for B7-H3 expression. The patients came from China, Japan, Austria, Norway, America and Germany and had been diagnosed with various cancers, including endometrial cancer, gastric carcinoma, cervical cancer, oral cancer, pancreatic cancer, osteosarcoma, esophageal cancer, colorectal cancer, breast cancer, hepatocellular carcinoma, ovarian carcinoma, lung cancer, prostate cancer, renal cell carcinoma (RCC), urothelial carcinoma of the bladder (UCB) and gallbladder cancer. Seven studies examined Caucasians, and 21 examined Asians. Twenty-six studies reported OS, 4 studies reported PFS, and 4 studies reported RFS. HRs and 95% CIs were directly reported in 15 studies and were calculated from survival curves in the other 13 studies. Cut-off values were different among the studies. The main characteristics of our included studies are presented in Table 1.

**Table 1:** Main characteristics of the studies in this meta-analysis

Author	Year	Country	Cancer	Case number	No. of B7-H3(+)	Detection method	Cut-off	Source of HR	Outcome measures	Follow-up (months)
Xu et al. [6]	2010	China	NSCLC	102	71	IHC	NR	Reported	OS	15–50
Brunner et al. [13]	2012	Austria	Endometrial	99	29	IHC	IHS ≥ 9	SC	OS	57(24–89)
Wu et al. [12]	2006	China	Gastric	102	60	IHC	Average positive cell ratio > 20%	Reported	OS	NR
Huang et al. [16]	2016	China	Cervical	108	78	IHC	IRS ≥ 3	Reported	OS	36–96
Chen et al. [17]	2015	China	Oral	72	48	IHC	NR	SC	OS	NR
Xu et al. [18]	2013	China	Pancreatic	45	35	IHC	IRS > 3	SC	OS	29.6(8–64)
Wang et al. [19]	2013	China	Osteosarcoma	61	37	IHC	IRS > 3	SC	OS	60(24–87.60)
Wang et al. [20]	2016	China	Esophageal	66	44	IHC	IRS > 3	Reported	OS	NR
Chen et al. [21]	2015	China	Esophageal	174	97	IHC	H-score > 185	Reported	OS	NR
Mao et al. [22]	2013	China	Colorectal	98	45	IHC	Percentage of stained lymphocytes > 20%	SC	OS	NR
Martin Loos [23]	2009	Germany	Pancreatic	68	28	IHC	IRS > 3	SC	OS	23(2–44)
Maeda et al. [24]	2014	Japan	Breast	90	52	IHC	IRS > 3	Reported	OS/RFS	67(7.8–90.5)
Sun et al. [25]	2012	China	Hepatocellular	240	168	IHC	IRS: 2 and 3	SC	OS/RFS	39 (1.5–95.0)
Arigami et al. [26]	2011	Japan	Gastric	95	48	qRT-PCR	NR	Reported	OS	24 (1–74)
Zang et al. [15]	2007	USA	Prostate	803	746	IHC	NR	Reported	PFS	84(63.6–99.6)
Zang et al. [10]	2010	USA	Ovarian	93	41	IHC	IHS > 50	SC	OS	Low stage: 96(7.2–306) High stage: 12(4.8–94.8)
Chen et al. [27]	2014	China	Pancreatic	63	43	IHC	Stained tumor cells > 10%	SC	OS	NR
Jin et al. [28]	2015	China	NSCLC	110	60	IHC	NR	SC	OS	6–60
Liu et al. [29]	2012	Norway	Prostate	130	70	IHC	NR	Reported	PFS/RFS	84(14–279)
Mao et al. [30]	2014	China	NSCLC	128	89	IHC	IRS ≥ 2	Reported	OS	53.3(40.3–74)
Zhou et al. [31]	2014	China	Colorectal	104	59	IHC	NR	SC	OS	NR
Boorjian et al. [32]	2008	USA	UCB	318	222	IHC	Stained tumor cells > 10%	Reported	OS/PFS	≥ 120
Ingebrigtsen et al. [33]	2014	Norway	Colorectal	731	637	IHC	NR	SC	OS/RFS	116.4(62.4–207.6)
Luo et al. [34]	2017	China	Lung	46	37	IHC	Percentage of immunoreactivity > 30%	Reported	OS	NR
Song et al. [35]	2016	China	Esophageal	100	66	IHC	Staining score > 3	Reported	OS/PFS	36(5.2–96)
Fukuda et al. [36]	2016	China	RCC	181	90	ELISA	NR	Reported	OS	31(3–100)
Liu et al. [37]	2016	China	Gallbladder	126	84	IHC	Stained tumor cells > 10%	SC	OS	NR
Inamura et al.[38]	2017	Japan	Lung	270	86	IHC	Intensity 1 ≥ 50% or intensity 2 ≥ 10%	Reported	OS	NR

NSCLC: non-small cell lung cancer; UCB: urothelial carcinoma of the bladder; RCC: renal cell carcinoma; IHC: immunohistochemistry; ELISA: enzyme-linked immunosorbent assay; OS: overall survival; PFS: progression-free survival; RFS: recurrence-free survival; NR: no report; SC: survival curve; IRS: immunoreactivity score; IHS: immunohistochemical score; HR: hazard ratio; qRT-PCR: quantitative real-time fluorescence polymerase chain reaction.

### B7-H3 expression and OS

**Figure 2:**
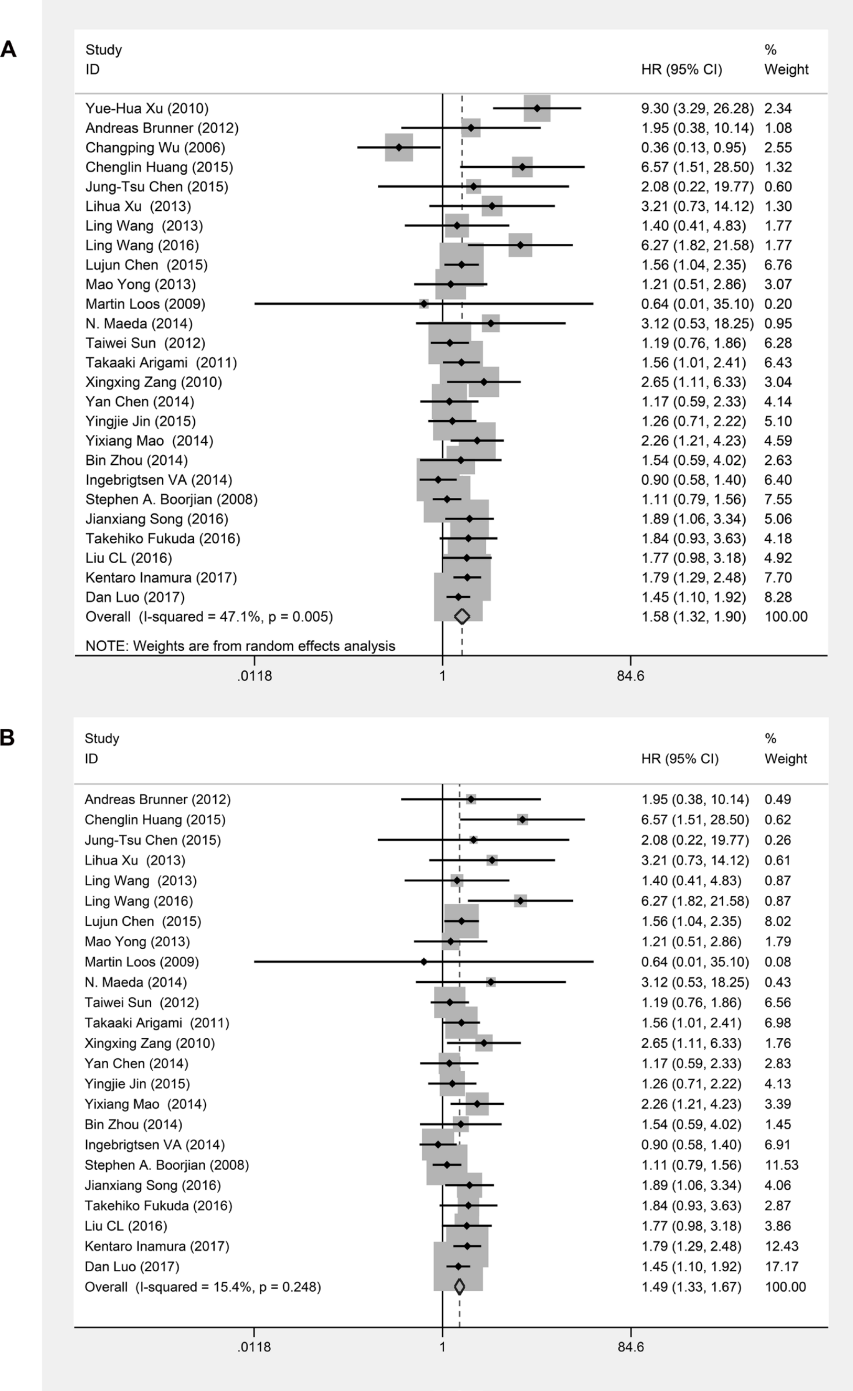
Forest plots of studies evaluating hazard ratios (HRs) of B7-H3 for overall survival (**A**) Summary of all twenty-six studies with regard to overall survival; the estimate was 1.58 (1.32–1.90) using a random effects model. (**B**) The exclusion of two studies [6, 12] resulted in no heterogeneity; the estimate was 1.49 (1.33–1.67) using a fixed effects model.

**Figure 3:**
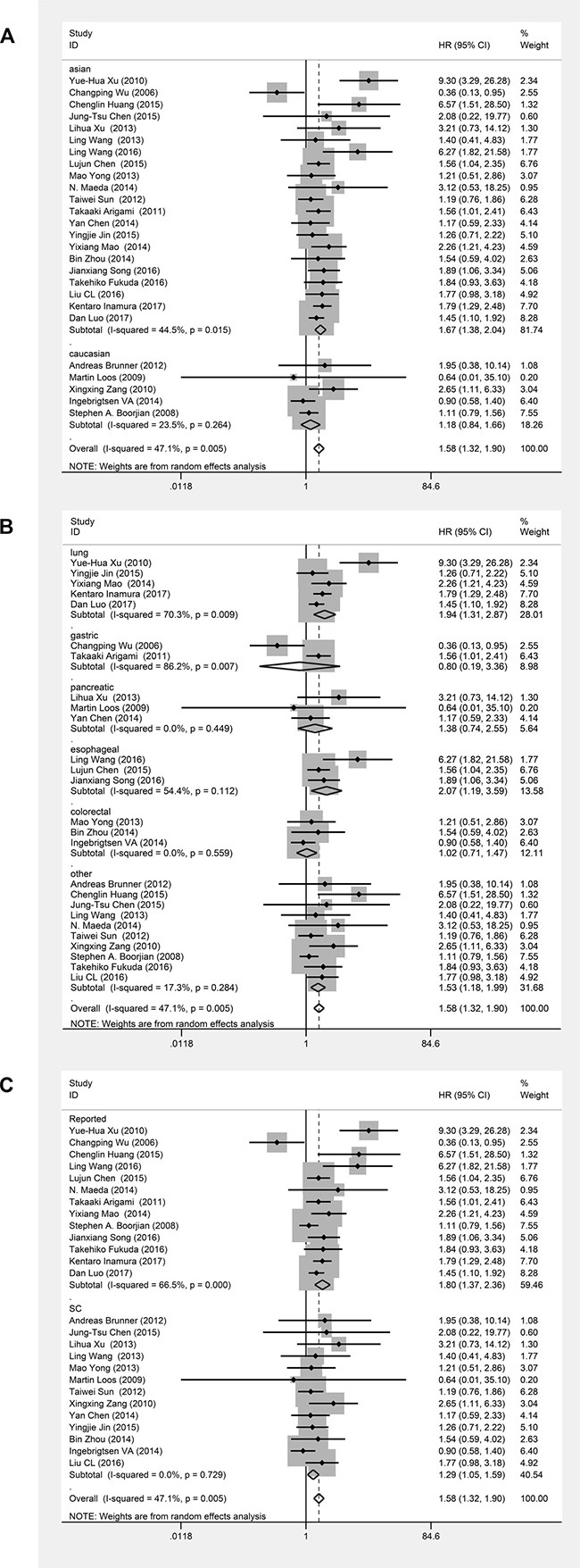
Forest plots of studies evaluating hazard ratios (HRs) of B7-H3 for overall survival in different subgroups (**A**) Subgroup analysis by ethnicity. (**B**) Subgroup analysis by cancer type. (**C**) Subgroup analysis by HR source.

Twenty-six studies evaluating 3690 patients were submitted to OS analysis. Because statistical heterogeneity was observed among these studies (*I*^2^ = 47.1%, *P*_heterogeneity_ = 0.005), we used a random effects model to pool their HRs. The main results of this meta-analysis are presented in Table 2. The pooled analysis showed that B7-H3 overexpression was significantly associated with worse OS in patients with solid tumors (pooled HR = 1.58; 95% CI = 1.32–1.90; *P* < 0.00001) (Figure 2). Excluding two studies [6, 12] resolved the heterogeneity (*I*^2^ = 15.4%, *P*_heterogeneity_ = 0.248) (Figure 2), but we did not find sufficient clinical heterogeneity to justify their exclusion. To explore the source of the identified heterogeneity in these studies, we performed subgroup analyses by ethnicity, cancer type and source of HR. The subgroup analysis for ethnicity showed that B7-H3 expression was significantly associated with worse OS in Asians (pooled HR = 1.67; 95%CI = 1.38–2.04; *P* < 0.00001), but not in Caucasians (pooled HR = 1.18; 95%CI = 0.84–1.66; *P* = 0.35); however, this difference was not statistically significant (*P*_D_ = 0.08) (Figure 3). The subgroup analysis based on tumor type showed that B7-H3 expression was significantly associated with worse OS for lung cancer (HR = 1.94; 95%CI = 1.31–2.87, *P* = 0.001), esophageal cancer (HR = 2.07; 95%CI = 1.19–3.59, *P* = 0.010) and other cancers including endometrial cancer, breast cancer, cervical cancer, oral cancer, osteosarcoma, hepatocellular cancer, ovarian cancer, RCC, UCB and gallbladder cancer (pooled HR = 1.53; 95% CI = 1.18–1.99; *P* = 0.001). However, there was no significant relationship between B7-H3 expression and OS in gastric cancer (HR = 0.80; 95% CI = 0.19-3.36; *P* = 0.759), pancreatic cancer (HR = 1.38; 95%CI = 0.74–2.55; *P* = 0.309), or colorectal cancer (HR = 1.02; 95%CI = 0.71–1.47; *P* = 0.901). Overall, the differences among cancer types were not statistically significant (*P*_D_ = 0.16) (Figure 3). Lastly, the subgroup analysis based on source of HR showed that differences between reported HRs and those based on survival curves were not statistically significant (*P*_D_ = 0.06) (Figure 3). To assess the stability of our results, we performed a sensitivity analysis by sequential omission of individual studies using a random effects model and found that the results were not clearly influenced by any single study (Supplementary Figure 1). We also evaluated the publication bias of all included studies using funnel plots and Egger's and Begg's tests. Visual inspection of the funnel plots (Supplementary Figure 2) revealed no evidence of publication bias, which was confirmed by Begg's test (*P* = 0.186) and Egger's tests (*P* = 0.100).

**Table 2:** Pooled HRs for overall survival and subgroup analysis of B7-H3 expression in cancer patients

	No. of study	No. of patients	Random effects model		Heterogeneity		*P_D_* value	*P* value
			Pooled HR	95%CI	*P* value	*I*^2^		
OS	26	3690	1.58	1.32–1.90	0.005	47.1%		< 0.00001
Ethnicity							0.08	
Caucasian	5	1309	1.18	0.84–1.66	0.264	23.5%		0.350
Asian	21	2381	1.67	1.38–2.04	0.015	44.5%		< 0.00001
Tumor type							0.16	
Lung	5	656	1.94	1.31–2.87	0.009	70.3%		0.001
Pancreatic	3	176	1.38	0.74–2.55	0.449	0		0.309
Gastric	2	197	0.80	0.19–3.36	0.007	86.2%		0.759
Colorectal	3	933	1.02	0.71–1.47	0.559	0		0.901
Esophageal	3	340	2.07	1.19–3.59	0.112	54.4%		0.010
Other	10	1388	1.53	1.18–1.99	0.284	17.3%		0.001
Source of HR							0.06	
Reported	13	1780	1.80	1.37–2.36	0.0003	66.5%		< 0.0001
SC	13	1910	1.29	1.05–1.59	0.729	0		0.014

OS: Overall survival; PFS: progression-free survival; RFS: recurrence-free survival; HR: hazard ratio; CI: confidence interval; PD: *P* value for subgroup difference; SC: survival curve.

### B7-H3 expression and PFS

**Figure 4:**
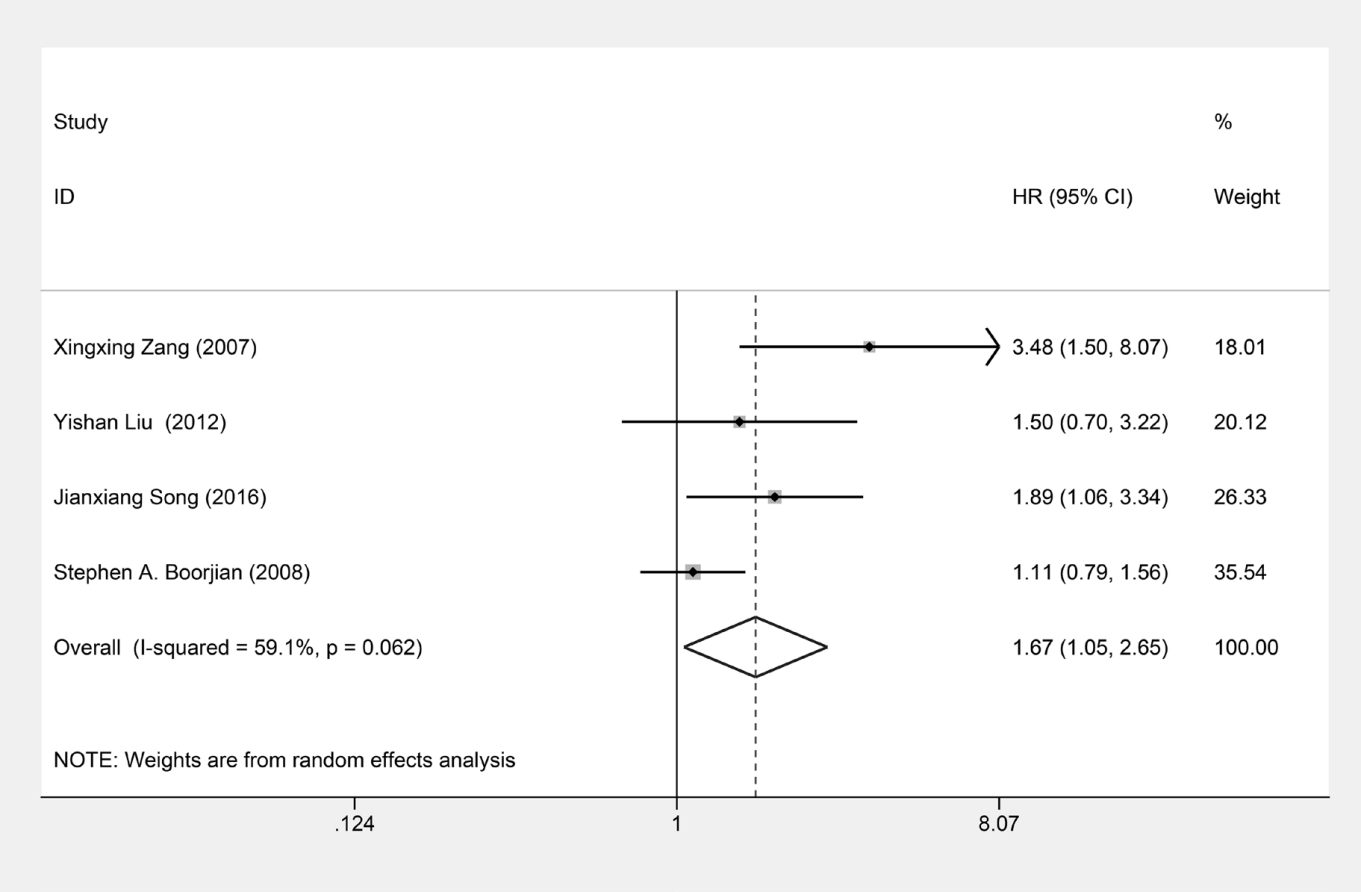
Forest plots of studies evaluating hazard ratios (HRs) of B7-H3 for PFS

Four studies evaluating 1351 patients underwent PFS analysis. Due to obvious statistical heterogeneity among these studies (*P*_heterogeneity_ = 0.062, *I*^2^ = 59.1%), we used a random effects model to pool their HRs. Our meta-analysis of these 4 studies revealed that B7-H3 expression was associated with poor PFS (HR = 1.67, 95%CI = 1.05–2.65, *P* = 0.031) (Figure 4).

### B7-H3 expression and RFS

**Figure 5:**
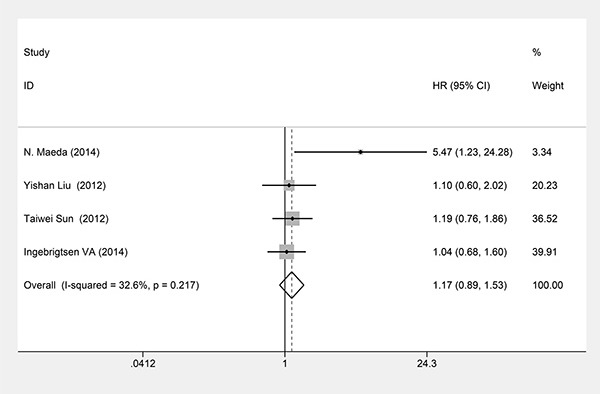
Forest plots of studies evaluating hazard ratios (HRs) of B7-H3 for RFS

Four studies evaluating 1191 patients underwent RFS analysis. A fixed effects model was used to pool their HRs because no obvious heterogeneity was observed among these studies (*P*_heterogeneity_ = 0.217; *I*^2^ = 32.6%). Our meta-analysis of these 4 studies showed that B7-H3 expression was not associated with RFS (HR = 1.17, 95%CI = 0.89-1.53, *P* = 0.267) (Figure 5).

## DISCUSSION

B7-H3, a member of the B7 family, was recently identified as an important co-stimulatory molecule that can be induced in B cells, T cells and DCs by a varity of inflammatory cytokines [2]. Although B7-H3 mRNA expression has been widely detected in human tissues, B7-H3 protein has rarely been found in normal tissues; instead, it is commonly detected on the surface of tumor cells [2]. Moreover, B7-H3 expression in tumor cells is associated with their migration, invasion and metastasis, ultimately leading to cancer progression [39, 40]. The precise physiological function of B7-H3 remains unclear. One early study showed that human B7-H3 functions as a co-stimulator of CD4+ and CD8+ T cells, promoting T cell proliferation and cytokine production *in vitro* [41]. However, most available data support the notion that B7-H3 inhibits T cell activation and proliferation [41–43]. Specifically, both the immunoglobulin-V-like and immunoglobulin-C-like forms of human B7-H3 inhibit T cell proliferation and reduce cytokine production in response to CD3/CD28-mediated co-stimulatory activation [43]. These findings indicate that B7-H3 can act as both a T cell activator and inhibitor.

By interfering with signaling pathways activated in non-immunological systems, B7-H3 promotes tumor progression, chemosensitivity and cancer metastasis. In the absence of lymphoid cells, Saran-mediated downregulation of B7-H3 significantly inhibited cancer cell migration and invasion, which suggests B7-H3 accelerates cancer progression via an unknown non-immunological mechanism [44]. B7-H3 expression correlates negatively with miR-29 levels, and the miR-29 binding site on B7-H3 mRNA is evolutionarily conserved, suggesting that a micro-regulatory mechanism influences B7-H3 expression profile at the mRNA and protein levels [45]. B7-H3 also appears to be involved in the Jak/Stat-3 pathway, which participates in multiple steps of metastasis, including invasion, survival, self-renewal, angiogenesis and immune evasion [46]. As Stat-3 is a convergence point for numerous signaling pathways and a valuable target for therapeutic intervention, a possible link between Stat3 and B7-H3 is of great interest.

Findings regarding the prognostic significance of B7-H3 in tumors have been controversial. Most studies suggested that high levels of B7-H3 expression in tumor cells were associated with disease progression and prognosis [8, 10, 11, 15, 25], but some reported no correlation between B7-H3 expression and tumor prognosis [47, 48]. Our results indicated that B7-H3 is highly expressed in 16 types of human cancer, and that high expression of B7-H3 is associated with poorer OS and PFS. However, our subgroup analysis showed that high B7-H3 expression was associated with poor OS in most cancers, but not gastric carcinoma, pancreatic cancer or colorectal cancer. The lack of consistency of these results may reflect differences in the methodologies used or differences in the function of B7-H3 in different cancers [16]. Although investigations have demonstrated the clinical significance of B7-H3 in different cancers, specific cognate receptors for B7-H3 have not been elucidated. The differing opposing effects of B7-H3 in different tumors may be associated with actions via different receptors, though this remains to be determined.

PD-L1 (B7-H1), another B7 family protein, suppresses immune responses by binding with PD-1 on T cells. Blocking the PD-1/PD-L1 pathway has led to significant clinical successes in a variety of cancers [49–51]. The expression pattern of B7-H3 and its functions differ from those of B7-H1. B7-H3 is mainly expressed on tumor cells and in the vasculature, while PD-L1 is expressed in immune cells, normal cells, and tumor cells [52]. B7-H1 expression in tumors often correlates with poor prognosis in cancer patients [53–55].

In addition to its immunological effects, B7-H3 can also promote cancer cell aggressiveness through various non-immunological pathways. Recently, antibodies targeting B7-H3 have been tested in several phase I/II clinical trials, and the results indicate B7-H3 is a promising target for future cancer immunotherapy. In addition, the relationship between B7-H1 and B7-H3 has been studied in some cancers. Chen et al. [27] showed that B7-H1 and B7-H3 promote pancreatic cancer progression, and their coexpression could be a valuable prognostic indicator. Mao et al. [30] found a positive correlation between expression of B7-H1 and B7-H3 in NSCLC and showed that expression of the two proteins is associated with poorer OS in patients with NSCLC. These findings suggest that treatments targeting B7-H3 and B7-H1 together may be a potential option for cancer immunotherapy.

There are several important implications of our meta-analysis. First, the results showed that B7-H3 overexpression is related to adverse OS and PFS in human solid tumors, indicating that B7-H3 may be a useful prognostic biomarker and therapeutic target for these tumors. Second, subgroup analysis based on cancer type showed that high B7-H3 expression is associated with poor OS in most cancers, but not some gastrointestinal cancers. The potential mechanism underlying this relationship is worthy of further study. Finally, B7-H3 has different functions in different cancers, which may reflect actions via different receptors. Inevitably, there are some limitations to our study. First, the numbers of studies and patients included in the PFS, RFS and OS subgroup analyses were relatively small, which could have resulted in heterogeneity. Second, the studies included in our analysis were all published in English, and thus some studies in other languages were omitted. Third, some studies did not provide accurate HRs for analysis, and we calculated HRs and 95% CIs based on survival curves, which resulted in small statistical errors. Finally, the cut-off values for B7-H3 expression differed among the included studies, which could have affected the stability of the results.

In conclusion, our results revealed that B7-H3 expression is associated with poor prognosis in most human solid tumors, suggesting that B7-H3 may be a useful prognostic biomarker and a promising new therapeutic target for solid tumors. In view of the limitations of our analysis, this conclusion should be considered cautiously. Some remaining questions and issues to be addressed in the future include identification of the exact receptors for B7-H3 and the establishment of standard assessment criteria. Accordingly, further prospective multicenter studies with larger samples will be needed to determine the role of B7-H3 in both the prognosis of and targeted therapy for various cancers.

## MATERIALS AND METHODS

### Literature search

The PubMed and Embase databases were comprehensively searched until 11 February 2017. In this meta-analysis, the Preferred Reporting Items for Systematic Reviews and Meta-Analyses (PRISMA) statement was followed [23]. The key words “B7-H3 OR CD276 OR B7 homolog 3 protein OR B7H3” AND “cancer OR tumor OR tumour OR neoplasm or carcinoma” AND “prognosis OR prognostic OR outcome OR survival OR fellow-up” were used to identify eligible studies. To identify additional potential studies, the reference lists of retrieved studies and reviews were also searched manually.

### Eligibility criteria

Studies were included in our meta-analysis if they met the following criteria: (1) studies focusing on B7-H3 expression and survival outcome, and (2) studies published as a full paper in English. Studies were excluded according to the following criteria: (1) reviews, letters, case reports, conference abstracts and laboratory studies; (2) studies with duplicate data or repeated analysis; (3) studies lacking sufficient information for further analysis; and (4) studies of hematological malignancies (because these cancers have unique mechanisms for tumorigenesis and metastasis). PubMed and Embase were searched independently by two authors (X. Zhang and C. Fang). For dual publications, the most detailed and informative study was selected for inclusion in the meta-analysis; unpublished literature, conference proceedings, dissertations and trial registries were excluded.

### Data extraction and quality assessment

The following information was extracted independently by two investigators: first author's name, publication year, country of origin, cancer type, number of patients, gender and age of patients, detection method, cut-off value, statistical method for survival analysis, occurrence of B7-H3 expression, and HRs with corresponding 95% CIs for overall survival (OS), and/or progression-free survival (PFS), and/or recurrence-free survival (RFS). If a study reported results from both univariate and multivariate analyses, the data for the multivariate analysis were selected. If HRs and CIs were not reported, the outcomes of interest were independently calculated using Kaplan-Meier curves by two investigators according to methods proposed by Tierney et al. [56]. Both investigators compared their datasets and combined the results to yield a final dataset. Disagreements were resolved in a consensus meeting.

Each of the 28 eligible studies was evaluated for methodological quality according to the Newcastle-Ottawa Quality Assessment Scale (NOS) [57]. The mean value of all included studies was a score of 6.89 (ranging from 5 to 8) (Supplementary Tables 1 and 2), indicating high quality and good methodology. Therefore, all studies were included in the next meta-analysis.

### Statistical analysis

HRs and corresponding 95% CIs obtained from the selected studies were used to calculate pooled HRs. Statistical heterogeneity was tested using the chi-square test, and the *I*^2^ statistic was used to assess the extent of statistical heterogeneity. If the *P* value was more than 0.1 for the chi-square test AND *I*^2^ was lower than 50%, indicating low heterogeneity, then a fixed effects model (the Mantel–Haenszel method) was used to calculate the pooled effect. Otherwise, a random effects model (the DerSimonian and Laird method) was used. Subgroup analyses were performed to explore the source of any identified heterogeneity, and sensitivity analyses were used to confirm the stability of the pooled results and to identify possible explanations for the observed heterogeneity. Publication bias was depicted by funnel plots and statistically assessed by Begg's and Egger's tests (with *P* < 0.05 indicating significant publication bias). Statistical analysis was performed using STATA 12.0 software. A *P* value of less than 0.05 was defined as statistically significant. All *P* values were two-sided.

## SUPPLEMENTARY MATERIALS FIGURES AND TABLES



## Abbreviations

OS: overall survival
PFS: progression-free survival
RFS: recurrence-free survival
HR: hazard ratio
CI: confidence interval
NSCLC: non-small-cell lung cancer
UCB: urothelial carcinoma of the bladder
RCC: renal cell carcinoma
IHC: immunohistochemistry
IRS: immunoreactivity score
IHS: immunohistochemical score
qRT-PCR: quantitative real-time fluorescence polymerase chain reaction
ELISA: enzyme-linked immunosorbent assay
NR: no report
SC: survival curve
PD: *P* value for subgroup difference
NOS: Newcastle-Ottawa Quality Assessment Scale
