# Supplementary material for: Prognostic value of B7-H3 expression in patients with solid tumors: a meta-analysis
Source: Oncotarget. 2017 Sep 21;8(54):93156–67. doi: 10.18632/oncotarget.21114 (PMC5696251; doi:10.18632/oncotarget.21114)
Supplement: Supplementary file 1 [file oncotarget-08-93156-s001.pdf]

Prognostic value of B7-H3 expression in patients with solid tumors: a meta-analysis

SUPPLEMENTARY MATERIALS

Supplementary Table 1: Newcastle-Ottawa Scale (NOS) for quality assessment in meta-analysis

**Selection**
1) Representativeness of the exposed cohort
a) truly representative of the average (describe) in the community★
b) somewhat representative of the average in the community★
c) selected group of users (e.g., nurses, volunteers)
d) no description of the derivation of the cohort
2) Selection of the non-exposed cohort
a) drawn from the same community as the exposed cohort★
b) drawn from a different source
c) no description of the derivation of the non-exposed cohort
3) Ascertainment of exposure
a) secure record (e.g., surgical records)★
b) structured interview★
c) written self-report
d) no description
4) Demonstration that outcome of interest was not present at start of study
a) yes★
b) no
**Comparability**
1) Comparability of cohorts based on the design or analysis
a) study controls for (select the most important factor)★
b) study controls for any additional factor★(This criterion could be modified to indicate specific control for a second important factor.)
**Outcome**
1) Assessment of outcome
a) independent blind assessment★
b) record linkage★
c) self-report
d) no description
2) Follow-up was long enough for outcomes to occur
a) yes (select an adequate follow-up period for the outcome of interest)★
b) no
3) Adequacy of follow-up of cohorts
a) complete follow-up - all subjects accounted for★
b) subjects lost to follow-up unlikely to introduce bias - small number lost – >75% (select an adequate %) or a description of those lost★
c) follow-up rate < 25% (select an adequate %) and no description of those lost
d) no

A study can be awarded a maximum of one star for each numbered item within the Selection and Outcome categories. A maximum of two stars can be given for Comparability.

Supplementary Table 2: Quality assessment of the 28 included studies with Newcastle-Ottawa Scale

Author	Year	Country	Cancer	Selection	Comparability	Outcome	NOS
Xu et al. [6]	2010	China	NSCLC	★★★	★★	★★	7
Brunner et al. [13]	2012	Austria	Endometrial	★★★	★★	★★	7
Wu et al. [12]	2006	China	Gastric	★★★	★★	★★	7
Huang et al. [16]	2016	China	Cervical	★★★	★★	★★★	8
Chen et al. [17]	2015	China	Oral	★★★	★	★★	6
Xu et al. [18]	2013	China	Pancreatic	★★★	★★	★★	7
Wang et al. [19]	2013	China	Osteosarcoma	★★★	★★	★★	7
Wang et al. [20]	2016	China	Esophageal	★★★	★★	★★★	8
Chen et al. [21]	2015	China	Esophageal	★★★	★★	★★	7
Mao et al. [22]	2013	China	Colorectal	★★★	★	★★	6
Martin Loos [23]	2009	Germany	Pancreatic	★★★	★	★★	6
Maeda et al. [24]	2014	Japan	Breast	★★	★★	★	5
Sun et al. [25]	2012	China	Hepatocellular	★★	★★	★★	6
Arigami et al. [26]	2011	Japan	Gastric	★★★	★★	★★★	8
Zang et al. [15]	2007	USA	Prostate	★★★	★★	★★★	8
Zang et al. [10]	2010	USA	Ovarian	★★★	★	★★★	7
Chen et al. [27]	2014	China	Pancreatic	★★★	★	★★★	7
Jin et al. [28]	2015	China	NSCLC	★★★	★★	★	6
Liu et al.[29]	2012	Norway	Prostate	★★★	★★	★★★	8
Mao et al. [30]	2014	China	NSCLC	★★★	★★	★★★	8
Zhou et al. [31]	2014	China	Colorectal	★★★	★★	★★	7
Boorjian et al.[32]	2008	USA	UCB	★★★	★★	★★★	8
Ingebrigtsen et al.[33]	2014	Norway	Colorectal	★★	★	★★	5
Luo et al.[34]	2017	China	Lung	★★	★★	★★	6
Song et al.[35]	2016	China	Esophageal	★★★	★★	★★	7
Fukuda et al.[36]	2016	China	RCC	★★★	★★	★★	7
Liu et al.[37]	2016	China	Gallbladder	★★★	★	★★	6
Inamura et al.[38]	2017	Japan	Lung	★★★	★★	★★	7

NOS: Newcastle-Ottawa Scale; NSCLC: non-small cell lung cancer; UCB: urothelial carcinoma of the bladder; RCC: renal cell carcinoma.

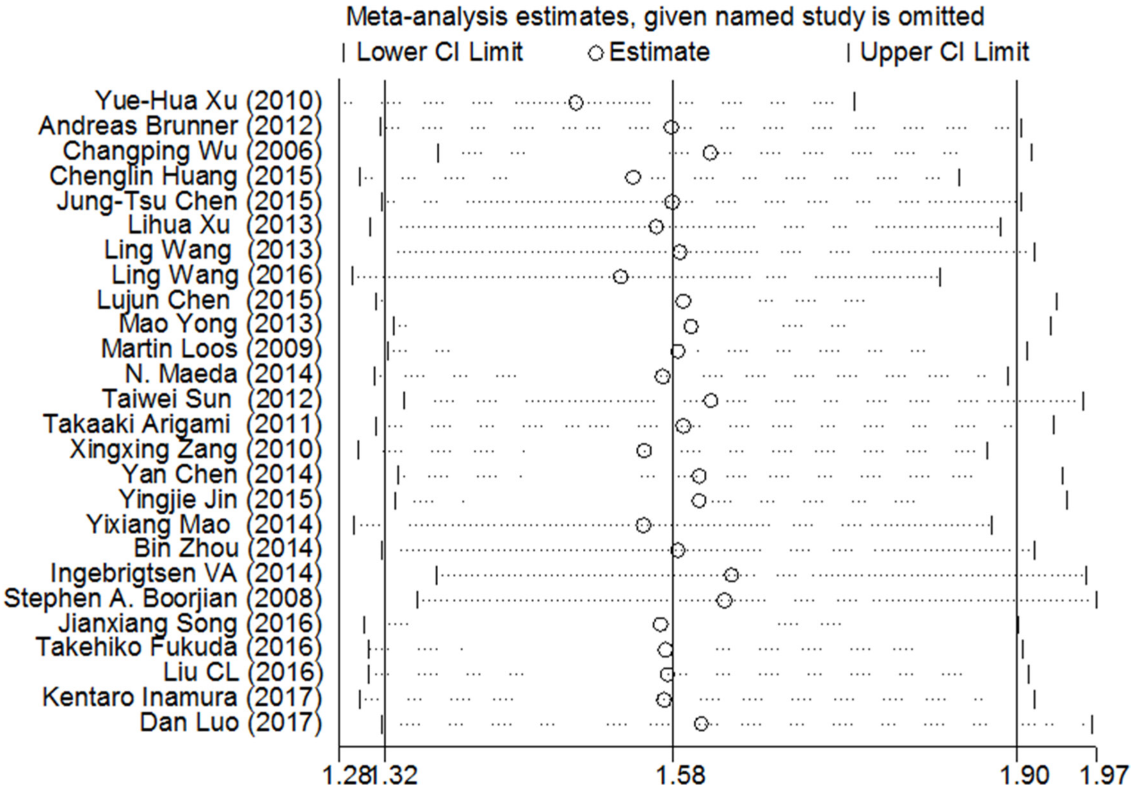

Supplementary Figure 1: Sensitivity analysis based on stepwise omission of one study at a time for overall survival.

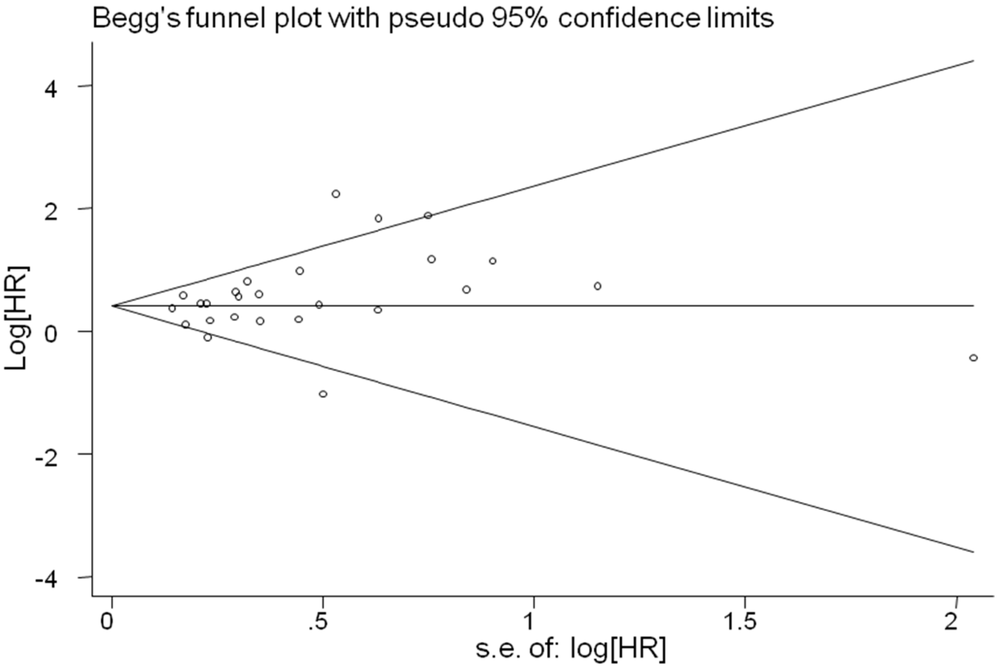

Supplementary Figure 2: Begg's funnel plots for all included studies reporting OS. Visual inspection of the plots did not identify substantial asymmetry**.**
